# Qualitative evaluation in nursing interventions—A review of the literature

**DOI:** 10.1002/nop2.519

**Published:** 2020-06-02

**Authors:** Kristine Rørtveit, Britt Saetre Hansen, Inge Joa, Kirsten Lode, Elisabeth Severinsson

**Affiliations:** ^1^ Department of Research, Nursing and Healthcare Research Group Stavanger University Hospital Stavanger Norway

**Keywords:** clinical nursing, implementation research, interventions, literature review, nurses, nursing, qualitative evaluation

## Abstract

**Aim:**

To identify and synthesize qualitative evaluation methods used in nursing interventions.

**Design:**

A systematic qualitative review with a content analysis. Four databases were used: MEDLINE, PsycINFO, Embase and CINAHL using pre‐defined terms. The included papers were published from 2014–2018.

**Methods:**

We followed the guidelines of Dixon‐Woods et al., Sandelowski and Barroso, the Critical Appraisal Skills Programme qualitative checklist and The Confidence in the Evidence from Reviews of Qualitative Research Approach.

**Results:**

Of 103 papers, 15 were eligible for inclusion. The main theme *Challenging complexity by evaluating qualitatively* described processes and characteristics of qualitative evaluation. Two analytic themes emerged: Evaluating the implementation process and Evaluating improvements brought about by the programme.

**Conclusion:**

Different qualitative evaluation methods in nursing are a way of documenting knowledge that is difficult to illuminate in natural settings and make an important contribution when determining the pros and cons of an intervention.

## 1 INTRODUCTION

During the last decade, there has been an ongoing discussion on the topic of developing and evaluating complex nursing interventions. Nursing interventions can be evaluated qualitatively, as this method enhances the significance of clinical trials and emphasizes the distinctive work and outcomes of nursing care (Sandelowski, 1996). However, there are few examples of detailed methodological strategies for doing so (Schumacher et al., 2005). Evaluation is a positive pursuit as it provides an organization with knowledge of how to improve or verify the value of services and how to determine which elements are strong and which are in need of improvement (Stufflebeam & Shinkfield, 2007). Nurses should therefore develop and implement strategies aimed at creating professional practice, and furthermore, such strategies should include designing and implementing performance measurement systems (McDavid & Huse, 2006). Morse, Penrod, and Hupcey (2000) describes Qualitative Outcome Analysis (QOA) as a method for qualitatively identifying intervention strategies and evaluating the implementation outcomes of patient‐oriented interventions.

### 1.1 Background

Clinical nursing is complex, and nurses need to understand the complexity of evaluation to improve their practice. The term “complex intervention” is widely used in the academic health literature to describe both health service and public health interventions. Complex interventions are defined as consisting of several components, which can act either independently or interdependently (Campbell et al., 2007; Mohler, Bartoszek, Kopke, & Meyer, 2012, p. 455). A complex intervention is characterized by several interacting components in several dimensions such as the behaviour required by the persons involved, the number of groups or levels in the organization, variability of outcomes and/or the degree of intervention flexibility (Craig et al., 2008).

The choice of evaluation method must be determined by its appropriateness for the purpose and intended use (Patton, 2015). Qualitative methods provide those who make decisions about the follow‐up of an intervention with access to a deeper understanding of the participants' experiences and perceptions of the intervention that goes beyond numbers and statistics (Patton, 2015). There are few studies about nursing intervention evaluation methods that describe the formal documentation of the content and delivery of a specific intervention in greater detail (Michie, Fixsen, Grimshaw, & Eccles, 2009) or factors that influence improvement in clinical nursing. Michie et al. (2009, p. 3) describe eight aspects that are essential in healthcare implementation: the content of the intervention, characteristics of those delivering the intervention, characteristics of the recipients, characteristics of the setting, the mode of delivery, the intensity (e.g. contact time), the duration (e.g. number of sessions over a given period) and adherence to delivery protocols. This is in accordance with Craig et al. (2008), who argue for several aspects necessary of development and evaluation: a good theoretical understanding, implementation problems, level processes, the range of measures and strict fidelity. Thus, we expand on the existing knowledge of complex interventions by searching for studies using qualitative evaluation methods to demonstrate a variety of methods used in relation to nursing evaluation and in the following we identify and synthesize the qualitative evidence of which research methods are applied when nursing interventions are evaluated.

## 2 THE REVIEW

### 2.1 Aim

To identify and synthesize qualitative evaluation methods used in nursing interventions. The review question addressed was: What characterizes the qualitative methods applied in evaluating the implementation of nursing interventions and improvements?

### 2.2 Design

A systematic literature review was conducted (Dixon‐Woods, Agarwal, Jones, Young, & Sutton, 2005; Hansen et al., 2012) on qualitative studies providing knowledge methods used in qualitative evaluation in the clinical nursing field. The qualitative review guidelines for assessing the quality of evidence presented by Sandelowski and Barroso (Sandelowski & Barroso, 2007) were adhered to.

### 2.3 Search method

A systematic search was carried out in MEDLINE, PsycINFO and Embase in October 2018. An additional search was performed in CINAHL to identify articles with nursing perspectives. Articles published in the previous 5 years (from January 2014) were included. The following search terms were used: qualitative evaluation, method* or tool* or model* or process* or strateg* or criteria or plan*, nurs* and implement* or improve* or intervention* or practice* or programme, patient*.

### 2.4 Search outcomes

The initial search revealed 103 articles, of which 40 were excluded due to being duplicates, 13 for not using a qualitative research method and 12 for other reasons such as not being performed by nurses, not involving nursing in the intervention or not involving patients. Appendix I shows the details and describes the identification process in accordance with PRISMA (Moher, Liberati, Tetzlaff, & Altman, 2009).

### 2.5 Inclusion and exclusion criteria

The searches were limited to peer‐reviewed journal articles in the English language on qualitative research methods applied in the evaluation of nursing interventions for adult patients (<18+ years) published within the previous 5 years. The narrow inclusion criteria stipulated articles focusing on nursing interventions in the clinical context and were from different hospital settings and community care. Articles on the family perspective, students' perspective and those employing mixed methods were excluded.

A total of 15 articles fulfilled the narrow inclusion criteria and were deemed appropriate for the review in addition to being relevant for illuminating the topics addressed by the review question. The Preferred Reporting Items for Systematic Reviews and Meta‐Analyses (Moher et al., 2009; PRISMA, 2018) and the Critical Appraisal Skills Programme (CASP, 2018) were used to structure the review process. The PRISMA flow diagram shows the selection process (Appendix I).

### 2.6 Quality appraisal and data extraction

Each article was initially critically appraised by two reviewers independently followed by a discussion among all five nurse researchers who finally reached consensus. All the included articles were quality checked in accordance with the checklist (CASP, 2018), see Appendix II and were sorted by study aim, intervention and context, method, results, qualitative evaluation and why it was performed as shown in Table 1.

**Table 1:** Overview of included papers

Authors/Year/Title	Aim	Intervention implemented and context	Method	Improvements	Evaluating implementation and improvements Reason for using qualitative evaluation and interpretation
1. Baron et al. (2018) USA “Increasing the Connectivity and Autonomy of RNs with Low‐Risk Obstetric Patients”	To explore the perspectives of patients, RNs and other providers regarding a new pre‐natal connected care model for low‐risk patients	The RN led model. Pre‐natal care A new pre‐natal connected care model for low‐risk patients aimed at reducing in‐office visits and creating virtual patient—RN connections Design: RCT. Context: Obstetric division	Patients' perspective: individual interviews with *N* = 26; asynchronous online focus groups *N* = 15 Providers: Baseline: 6 physicians, 8 CMNs, 9 RNs Midpoint: 8 physicians, 2 RNs, 6 nurses, 1 nurse supervisor Study completion: 6 physicians, 9 CMNs. Semi‐structured interview guide based on evaluation framework RE‐AIM and normalization theory Thematic analysis	The RN led model increased patient satisfaction and gave RNs greater autonomy; patients valued connectedness with a small number of dedicated RNs and the ability to contact them as needed outside the office setting; physicians appreciated having more time to care for higher‐risk patients; RNs appreciated being able to work with a fuller scope in their practice	*Evaluating the implementation process* To explore in depth how various stakeholders viewed the role of Registered Nurses in a new model
2. Bolmsjo et al. (2014) Sweden “The use of drama to support reflection and understanding of the residents' situation in dementia care: a pilot study”	To explore the use of drama as a tool to support reflection among staff working in residential care for people with dementia	Drama as a tool in residential dementia care Context: Residential care	Nurse assistants' perspective: Observations and tape recordings from sessions One focus group interview with nurse assistants after the end of the intervention Analysis: Content analysis on the manifest level	Reflection about daily caring practice was stimulated; information about the purpose of the sessions is important; the research team must ensure the defined frames and conditions and have practical knowledge about caring for people with dementia; the management needs to be stable, committed and supportive	*Evaluating the improvements brought about by the programme* Qualitative evaluation of the programme consisting of three drama sessions with staff (*N* = 10 nurse assistants). Pilot study
3. Clignet et al. (2017) The Netherlands “A Qualitative Evaluation of an Inpatient Nursing Intervention for Depressed Elderly: The Systematic Activation Method”	To describe the evaluation of the implementation of a nursing intervention—the SAM	The Systematic Activation Method among in‐patients with late‐life depression Context: Four clinical units for old age psychiatry	Nurses' perspective: Four group interviews (12 nurses) Analysis: Thematic content analysis Quantitative method: questionnaire/nurses and questionnaire/patients *N* = 10 as well as the Beck Depression Inventory scale. The Minimal Mental State Examination constituted another part of study	The implementation process is complex; to perform thorough analysis before and during implementation of barriers and facilitators complexity of intervention, patient group, nurses, nurse–patient interaction, organizational factors; careful supervision and monitoring of the implementation process; active participation of management and multidisciplinary team	*Evaluating the implementation process* To find which implementation factors are most relevant to this population ‐ to identify facilitators and barriers relating to patient and nurse characteristics, as well as to contextual factors
4. Davisson and Swanson (2018) USA “Patient and Nurse Experiences in a Rural Chronic Disease Management Program: A Qualitative Evaluation”	To evaluate and improve the nurse‐led “Living Well” chronic disease management programme	A chronic disease management programme. The CCM was the guiding framework for an evaluation of the programme. Patient groups: heart failure, diabetes, COPD Context: Rural, critical access hospital	Patient perspective: Interviews with 6 rural, English‐speaking adults (65 years or older, with no severe cognitive impairment) with at least one chronic condition: Observations Analysis: Within and across case coding. Nurse perspective: Interviews with 2 nurse coordinators of the programme were not included in this study	The programme is important; lack of commitment to the programme; there is an overreliance on coordinators to manage all programme aspects; to achieve more efficient communication when identifying eligible patients; appropriate patient referral processes to the programme are important	*Evaluating the improvements brought about by the programme* What are the reasons for recruitment and retention problems? What elements of the programme are successful or need improvement?
5. Furler et al. (2014) Australia “Stepping up: a nurse‐led care model for insulin initiation for people with type 2 diabetes”	To describe the development and evaluation of a nurse‐led care model	A nurse‐led care model for insulin initiation Context: 4 GP practices	Patient and health care profession perspective: After 3 months: 10 participating patients joined in focus group interviews After 12 months: 12 patients, 7 GPs and 5 PNs participated in telephone interviews Analysis: Qualitative data were analysed thematically	Defining and legitimating new roles particularly for PNs; The importance of relational continuity between PN and patients; A long‐standing relationship with and knowledge of patients are essential for providing information, education and addressing concerns in a timely manner that suited patients	*Evaluating the improvements brought about by the programme* Process evaluation of the experiences of PNs, GPs and patients involved in the programme to find: how the programme integrated or caused tensions with routine care practice ‐explore enablers and facilitators, which has implications for sustainability and generalizability in practice
6. Graves et al. (2016) UK “Psychological skills training to support diabetes self‐management: Qualitative assessment of nurses' experiences”	Explores nurses' experience of training in six psychological skills to support patients' self‐management of type 2 diabetes	Psychological skills training (6 psychological skills) of primary care nurses to support the self‐management of patients with type 2 diabetes Context: GP surgeries in 5 rural boroughs	Nurses' perspective: Semi‐structured interviews with 9 nurses delivering the intervention and 7 nurses from the control intervention Analysis: Thematic framework analysis	Nurses felt they were overstepping their professional role when dealing with emotive consultations as they did not feel qualified and had to adjust their role to facilitate the use of the new skills; the skills felt valuable and transferable to primary care; they felt under supported by their practice and the research team. Positive impact: Patient empowerment. Negative impact: Patients' capacity to engage	*Evaluating the improvements brought about by the programme* Explore nurses' experiences of exercising the new skills To reveal mechanisms which hinder or promote implementation of the intervention according to the protocol
7. Hahne et al., (2017) Sweden “Changes in professionals' beliefs following a palliative care implementation programme at a surgical department: a qualitative evaluation”	To evaluate how the implementation of a combination of integration and consultation strategies, can change beliefs among professionals related to the implementation of palliative care in hospitals	Implementation of palliative care using a combination of integration and consultation strategies through an educational implementation strategy Context: Surgical department, palliative care	Nurse and physician perspectives: Before introducing the implementation strategy: 2 focus groups (FG 1: *N* = 3, FG 2: *N* = 3) 2 nurses, 1 physician After implementation: one focus group (*N* = 6) 5 nurses, 1 physician Analysis: qualitative systematic text condensation	Positive changes regarding palliative care in six out of seven areas were found: working methods in palliative care, team collaboration in palliative care, collegial support, discussions about diagnosis, symptoms at the end of life and the families of patients in palliative care. No change in team collaboration in palliative care was found	*Evaluating the implementation process* To identify specific contextual belief areas related to the implementation of palliative care in hospitals. The change in beliefs involved differences regarding surgical and palliative care
8. Halcomb et al. (2015) Australia “Process evaluation of a practice nurse‐led smoking cessation trial in Australian general practice: views of general practitioners and practice nurses”	To perform a process evaluation of a PN‐led smoking cessation intervention, RCT	A practice nurse‐led smoking cessation trial entitled Quit with PN. Free nicotine replacement therapy patches for 8 weeks were offered to participants and a 1‐day workshop for all participating PNs. Context: General practice	Nurses and general practitioners' perspectives: Semi‐structured telephone interviews with 15 GPs and 22 PNs allocated to the intervention arm (Quit with PN) Analysis: NVivo and thematic analysis	Results: The Quit with PN intervention was viewed positively. Most PNs were satisfied with the training and the materials provided. Barriers in integrating the PN role into the daily work of the practice: challenges in managing patient data, managing the workload and communication between GPs and PNs	*Evaluating the improvements brought about by the programme* The Quit with PN intervention was viewed positively. Some challenges in managing patient data, follow‐up and communication between GPs and PNs were identified
9. Hanifa et al. (2018) Denmark “Picking up the pieces: Qualitative evaluation of follow‐up consultations postintensive care admission”	To describe former intensive care patients' experiences of a nurse‐led consultation regarding symptoms of Postintensive Care Syndrome and to explore its benefits	A nurse‐led consultation 3 months postintensive care unit admission to help former intensive care patients cope with postintensive care syndrome and identify opportunities for further intervention. Context: After discharge from the Intensive Care Unit (ICU), former ICU patients	Patients' perspective: Focused ethnography combining observations and interviews. 10 patients participated in a 2‐part qualitative study: (a) an observational study of the current follow‐up consultation; (b) a semi‐structured interview based upon observations and statements arising during the initial consultation. Analysis: hermeneutic phenomenological approach	Content and setting of the consultation were of the utmost importance; revisiting the unit and experiencing the setting in person played a huge role in coping with postintensive care syndrome; involving relatives was essential, as they were an important part of the patient's rehabilitation	*Evaluating improvements brought about by the programme* Qualitative evaluation of a nurse‐led consultation 3 months after ICU stay
10. Helmle et al. (2018) Canada “Qualitative Evaluation of the Barriers and Facilitators Influencing the Use of an Electronic Basal Bolus Insulin Therapy Protocol to Improve the Care of Adult In‐patients With Diabetes”	Evaluation of an electronic basal bolus insulin therapy protocols' effect on workflow and practice and exploration of potential barriers and facilitators to its use	A new evidence‐informed electronic basal bolus insulin therapy protocol to improve diabetes care and practice. Context: 3 adult acute care facilities	Nurses, resident trainees and physicians' perspective: Semi‐structured focus groups with 9 multidisciplinary nursing staff (*N* = 22), resident trainees (*N* = 24) and attending physicians (*N* = 23) involved in the delivery of inpatient diabetes care. Analysis: content analysis approach	Themes including the impact of education, information technology/user interface, workflow, organizational issues and practices, and perceived outcomes	*Evaluating improvements brought about by the programme*
11. Hill et al. (2016) Australia “It promoted a positive culture around falls prevention': staff response to a patient education programme‐a qualitative evaluation”	To understand how staff responded to individualized patient fall prevention education, RCT	An individualized patient fall prevention education Context: Units providing elder care ranging from acute to rehabilitation, 5 different hospitals (public health)	Health care professionals' perspectives: A total of 5 focus groups were conducted at 5 different hospitals with 12 nurses, 3 senior clinical nurses, 12 allied health professionals, 1 medical doctor, 2 quality improvement staff Thematic analysis by means of NVivo	Education created a positive culture around fall prevention and facilitated teamwork, whereby patients and staff worked together to address fall prevention and developed increased knowledge and awareness about creating a safe ward environment; patients were more proactive and empowered to engage in fall prevention strategies	*Evaluating improvements brought about by the programme* Qualitative evaluation of staff response of educational program
12. Iyer et al. (2015) USA “A qualitative evaluation of capnography use in paediatric sedation: perceptions, practice and barriers”	To explore perceptions about and barriers to the use of capnography for procedural sedation	The use of capnography Context: Paediatric emergency department in an urban trauma centre	Nurses and physicians' perspective: Grounded theory approach. 5 paediatric emergency medicine professionals and 12 RNs from the paediatric emergency department participated in one‐to‐one interviews Analysis: Grounded Theory	Procedural sedation is safe and adverse events are rare; normal capnography readings reassured providers about the adequacy of ventilation; Knowledge and comfort varied and additional education and training were requested; use of sedation was infrequent; increased use in other paediatric populations	*Evaluating improvements brought about by the programme* Qualitative evaluation of the use of capnography
13. Kang et al. (2017) South Korea “Qualitative evaluation of a delirium prevention and management programme”	To evaluate a 3 months educational program for RNs' to improve knowledge and attitude in delirium care for hospitalized older adults with and without dementia	Educational programme in delirium care based on adult learning principles Context: 4 medical wards in a regional general hospital	Nurses' perspective: 12 Registered Nurses who participated in the educational programme took part in individual interviews. Analysis: Content analysis The programme was also evaluated quantitatively	Improved knowledge of and attitude towards the delirium care of hospitalized older adults with dementia and at risk of delirium; active learning in the programme facilitated the participants' learning processes; inadequate management support to apply their new knowledge in practice, included staff resources, policies and protocols	*Evaluating improvements brought about by the programme* To explore RNs' perceptions of the programme in depth and to examine the strengths and weaknesses of the programme
14. Luker et al. (2016) Australia, New Zealand, Scotland “Implementing a complex rehabilitation intervention in a stroke trial: a qualitative process evaluation of AVERT”	To better understand how the implementation of a rehabilitation intervention is experienced by the staff involved	Rehabilitation intervention Context: Acute stroke units	Health and nursing staff perspective: Semi‐structured phone, voice‐Internet, or face to face interviews of 53 health and nursing staff from 19 acute stroke units. Analysis: rigorous thematic analysis. Part of a quantitative study	Extra work but rewarding; Team practices changed; Challenges such as lack of established interdisciplinary teamwork and inadequate staffing levels at some sites; various organizational barriers, staff attitudes and beliefs, and patient‐related barriers; Enthusiastic team leadership was crucial to success	*Evaluating the implementation process* Qualitative process evaluation of the implementation of a rehabilitation intervention as experienced by the staff involved
15. Soderlund et al. (2016) Sweden “Conversations between persons with dementia disease living in nursing homes and nurses—qualitative evaluation of an intervention with the validation method”	To illuminate the actions and reactions of persons with DD in conversations with nurses during 1 year of VM training	VM training programme to facilitate nurses' communication with persons with DD, focusing on the fact that each person is unique. One year training programme Context: Nursing homes	Nurse‐person perspective: Naturalistic design. 4 persons with DD were involved in videotaped conversations (one‐to‐one) with four nurses. Analysis: qualitative analysis of visual data with focus on nursing skills in nurses' communication with persons with DD	Not treating the person like an adult is a barrier to communication; Allowing the person to choose the topic of communication is stimulating; talking about more than one topic at the same time; trying to talk more freely about what is on one's mind	*Evaluating improvements brought about by the programme* Qualitative evaluation of VM training programme To illuminate the actions and reactions of persons with DD living in nursing home with nurses who had taken part in the VM method training programme

Abbreviations: AVERT, A Very Early Rehabilitation Trial; CCM, chronic care model; CMNs, certified nurse midwives; COPD, chronic obstructive pulmonary disease; DD, dementia disease; GP, general practitioner; ICU, intensive care unit; PN, practice nurses; RCT, randomized controlled trials; RN, Registered Nurses; SAM, Systematic Activation Method; VM, validation method.

### 2.7 Review

The qualitative review adhered to (Sandelowski & Barroso, 2007). The analysis was performed by a thorough reading and rereading of the articles (Dixon‐Woods et al., 2006). The data were analysed stepwise following a manifest content analysis technique (Graneheim & Lundman, 2004). After each article had been thoroughly assessed, they were sorted and summarized. In the analysis process, the text describing the evaluation method was considered to constitute the meaning units (Graneheim & Lundman, 2004). The meaning units were then coded and thematized as groups of content that shared a similar meaning. The qualitative evaluation method was reflected on, discussed and finally formulated into one theme and three sub‐themes. The sub‐themes helped to describe the identified factors. The main theme and sub‐themes were created by abstraction of the categorized meaning units in a process involving all the authors. Various alternatives were discussed by the authors to reach consensus on the sorting and labelling. Research Ethics Committee approval was not required.

## 3 RESULTS

### 3.1 Characteristic of the studies

Of 103 papers, 15 were eligible for inclusion (Baron et al., 2018; Bolmsjo, Edberg, & Andersson, 2014; Clignet, van Meijel, van Straten, & Cuijpers, 2017; Davisson & Swanson, 2018; Furler et al., 2014; Graves, Garrett, Amiel, Ismail, & Winkley, 2016; Hahne, Lundstrom, Levealahti, Winnhed, & Ohlen, 2017; Halcomb et al., 2015; Hanifa, Glaeemose, & Laursen, 2018; Helmle, Edwards, Kushniruk, & Borycki, 2018; Hill et al., 2016; Iyer, Koziel, & Langhan, 2015; Kang, Moyle, Cooke, & O'Dwyer, 2017; Luker et al., 2016; Soderlund, Cronqvist, Norberg, Ternestedt, & Hansebo, 2016) and the PRISMA flow diagram shows the selection process (Appendix I I). The 15 included articles are presented in Table 1, and there is an example of the questions, while Appendix II contains the criteria from the CASP checklist. Overall, we found that the included articles had a high score, although adequate consideration of the relationship between the researcher and participants was lacking in several articles. The most common methodology was interviews, either individual or in focus groups. Educational programmes were the most frequently used intervention, and thematic analysis was the methodology most often employed. Two analytic themes emerged: Evaluating the implementation process and Evaluating improvements brought about by the programme (Table 2). One main theme was developed from this process: *Challenging complexity by evaluating qualitatively*. The main theme outlined how the design of an evaluation of the intervention was influenced by the inherent complexity.

### 3.2 Theme 1: Evaluating the implementation process

This theme described the different types of evaluation design used in the implementation processes, data characteristics and context as well as types and models of analysis.

#### 3.2.1 Different types of designs

The theme *different types of design* was based on the sub‐category *aims and types of data*, where we found a great variation in the descriptions employed. Some of the studies aimed to report and evaluate the intervention from the *staff perspectives*, while others described and evaluated the *patients' perspectives* or reported *both perspectives* (Baron et al., 2018). Changes associated with the interventions were examined by some, while others explored experiences of care or evaluated experiences and perceptions of an intervention. Several of the *aims* concerned contributing to a *deeper knowledge* in staff members' daily practice; to *better understand* their experiences and *explore perceptions and perspectives* of an intervention (Graves et al., 2016; Iyer et al., 2015; Luker et al., 2016). Other examples from staff members' perspectives aimed at exploring the use of drama as a tool (Bolmsjo et al., 2014) or developing a model of care (Furler et al., 2014).

Examples of more detailed formulations of the aims were: to improve a programme (Davisson & Swanson, 2018), evaluate a programme's impact on staff's knowledge and attitude (Kang et al., 2017) or to evaluate effect on practice (Helmle et al., 2018). Some studies aimed to evaluate the effect of workflow and practice and to examine the strength and weaknesses of a programme (Helmle et al., 2018; Kang et al., 2017). The various aims demonstrated ways of detecting the knowledge sought by the evaluation, and all of them were grounded in a design with a qualitative tradition.

The *types of data* pointed to a variety of different data collection methods in qualitative evaluations. They all included some form of *in‐depth interviews,* and *semi‐structured interviews* were common (Baron et al., 2018; Graves et al., 2016; Halcomb et al., 2015; Hanifa et al., 2018; Helmle et al., 2018; Luker et al., 2016). Several studies employed one or several *focus group interviews* (Baron et al., 2018; Bolmsjo et al., 2014; Furler et al., 2014; Hahne et al., 2017; Hill et al., 2016), and there were several examples of *combined methods,* such as evaluation interviews, focus group and telephone interviews (Furler et al., 2014), telephone interviews, voice Internet or face to face (Luker et al., 2016), observations and tape recordings during sessions, focus group interview and written reflections (Bolmsjo et al., 2014). Other examples of data collection were related to the time the data were collected: for instance, a process evaluation conducted by means of qualitative data collected 3 and 12 months postintervention (Furler et al., 2014).

We found no explicit explanations of or reflections on why the specific design was chosen in any of the articles, although an implicit understanding was present.

#### 3.2.2 Data characteristics and context

The *different data and context of problems* pertaining to the evaluations varied, illuminating the range of fields where qualitative evaluation methods can be valuable in an implementation process. This category describes the types of setting, problem and diagnosis. The data represent a variety of clinical settings and were collected in natural healthcare contexts. Several evaluations were performed in a typical somatic hospital setting such as acute stroke, paediatric, surgical ICU or obstetric departments (Baron et al., 2018; Hahne et al., 2017; Hanifa et al., 2018; Iyer et al., 2015; Luker et al., 2016). In addition, community settings such as elder and dementia care (Bolmsjo et al., 2014) and diabetes care (Furler et al., 2014) were evaluated. The settings of the various studies represented different clinical contexts; acute and emergency care, long‐term care and general practice, community settings and hospital units, all of which were representative of a *complex intervention*.

#### 3.2.3 Types and models of analysis

All the reviewed articles presented established *models of analysis* in the methodological section, which provided a detailed description of how the analysis was performed. In addition to traditional qualitative analysis, the articles described more advanced models of analysis such as thematic content analysis, the hermeneutic phenomenological approach, grounded theory, conventional inductive content analysis (Clignet et al., 2017; Hanifa et al., 2018; Iyer et al., 2015) and several forms of content analysis. This summary shows the variety of methods that can be chosen.

The question of whether the evaluation of the detailed intervention was performed inductively or deductively was addressed in some of the articles (Bolmsjo et al., 2014; Furler et al., 2014; Iyer et al., 2015; Luker et al., 2016) but only when explicitly stating that an inductive approach was used. In several of the studies, it seemed as if the reason for choosing a qualitative design was to capture the complexity.

### 3.3 Theme 2: Evaluating improvements brought about by the programme

This theme analyses the improvements as they were described in the studies that is, the intervention process; types of intervention and characteristics of those who deliver the intervention. The implementation processes were complex, but the qualitative analysis and highlights of the articles made the outcome of the interventions visible.

#### 3.3.1 Clinical benefits

The outcomes were connected to the *clinical benefits*. For instance, important themes that provided more insight into clinical implementation in complex care settings were described (Luker et al., 2016). These included the fact that the implementation required extra work but was rewarding; that team practices changed; that challenges such as the lack of established interdisciplinary teamwork and inadequate staffing levels arose at some sites; that there were various organizational barriers, the impact of staff attitudes and beliefs and patient‐related barriers; and that enthusiastic team leadership was crucial for success. Another example was described by Clignet et al. (2017), who studied the implementation process to find which implementation factors are most relevant to this population and to identify facilitators and barriers relating to the characteristics and contextual factors of patients and nurses (Clignet et al., 2017).

One study revealed that although the participants considered the intervention safe, they did not use it (Iyer et al., 2015). Another result revealed that the intervention could be a means to enhance reflection on daily caring practice among nursing staff (Bolmsjo et al., 2014), while one found that the RN led model increased patient satisfaction and gave RNs greater autonomy (Baron et al., 2018). Positive changes in palliative care were described, such as working methods, team collaboration, collegial support, discussions about diagnosis, symptoms at the end of life and the patient's family members (Hahne et al., 2017). Involving relatives was found to be essential in the rehabilitation of former intensive care patients (Hanifa et al., 2018). A study on fall prevention described that an education programme created a positive culture whereby patients and staff worked together to address falls prevention and gained awareness about creating a safe ward environment (Hill et al., 2016).

The study on a 1‐year training programme on validation communication for nurses described the reactions of patients with dementia and found that actions such as not treating the patient as an adult constitute a barrier to communication or talking more freely about what is on one's mind (Soderlund et al., 2016). In one study on a care model for insulin initiation, a long‐standing relationship with and knowledge of patients was described as essential for providing information, education and addressing concerns in a timely manner that suited patients (Furler et al., 2014). In a study on psychological skill training to support patients with diabetes‐2, nurses described a sense of overstepping their professional role when dealing with emotive consultations as they did not feel qualified and had to adjust their role to facilitate the use of the new skills (Graves et al., 2016).

One article described how important the chronic disease management programme was despite a lack of commitment to it. There was an overreliance on coordinators to manage all aspects of the programme and that more efficient communication was necessary when identifying appropriate patients to refer to the programme (Davisson & Swanson, 2018). We found that the outcome in all articles was of benefit to clinical practice, despite the fact that no numerical or statistical data were presented.

#### 3.3.2 Types of intervention

As we did not limit the type of clinical implementation when selecting the articles, the *types of intervention* included in this review were broad. The models and programmes implemented were thoroughly described in the articles. Most of the interventions comprised programmes *involving models or guidelines* such as drama as a tool (Bolmsjo et al., 2014) and the care model for insulin initiation (Furler et al., 2014). Few of the studies described procedures in detail, with the exception of one study on sedation during the capnography procedure (Iyer et al., 2015). The patient nurse perspective and the intensity and duration of the intervention were thoroughly described in each article. The *utility* of the intervention and *why* such interventions were necessary were also outlined.

#### 3.3.3 Characteristics of those who deliver the intervention

In the articles, several professional categories were involved in the implementation process and described in accordance with the mode of delivery and the organizational level of the intervention. Some articles involved only nursing staff, either with *one specified nursing specialty* or with *different types of nursing specialty*. Other articles described a *multidisciplinary combination* of nurses and other professionals, for instance physiotherapists, personal trainer assistants and speech pathologists, paediatric emergency medicine professionals, general practitioners (GPs) and endocrinologists (Furler et al., 2014; Iyer et al., 2015; Luker et al., 2016).

The organizational level did not vary as much as the professional categories. However, some of the articles combined more than one unit, for instance several clinical units for old age psychiatry, adult care facilities or different medical wards in a regional hospital (Clignet et al., 2017; Helmle et al., 2018; Kang et al., 2017). The evaluation studies were performed in their natural setting, and the mode and description of the delivery and the organizational level of the intervention provided important information that illuminated the complexity of the actual clinical setting.

## 4 DISCUSSION

The aim of this review was to identify and synthesize qualitative evaluation methods used in nursing interventions, and the review question was *What characterizes the qualitative methods applied in evaluating the implementation of nursing interventions and improvements?* This review illuminates how evaluating the implementation of nursing interventions and improvements i*s challenging because of the complexity involved,* which is described by the variety of different methods included in the qualitative evaluation of interventions. The review states that different perspectives of the qualitative evaluation designs highlight the variation and benefits of such evaluation.

The implementation process perspective illuminates the obvious reasons for performing the actual evaluation based on the design, the problems revealed, and the analysis methods employed. The evaluation perspective demonstrates how improvements based on concrete benefits are crucial. The actual evaluation of the intervention shows the importance of thorough descriptions of the implementation strategies, those who deliver the intervention and the level of the activity.

From the methodological perspective, we were surprised to detect such different modes and creative ways of handling the need to evaluate complex situations in clinical practice. Although several of the included articles aim to *explore*, we hold that the concept *exploring experiences* is continuous and needs to be considered a little further. According to van Manen, qualitative methods *explore* a variety of issues such as empirical questions or perceptions (p. 811). Qualitative methodology focuses on individuals, and the clinical evaluations as unique examples are under the spotlight in the current review. Therefore, *What*‐questions are crucial as they provide insight. However, only a few articles explicitly aimed to gain insight. The concept *explore* is typically used in phenomenological approaches, but only one article in the present review claims to adopt a hermeneutic phenomenological approach; as the authors study the patients' perspective they combine observations, interviews and a hermeneutic phenomenological approach to analyse the data (Hanifa et al., 2018). The original meaning of a phenomenon is captured by phenomenology; to bring *experience* we lived through to our awareness retrospectively; and to be able to reflect on the lived meaning of the experience (van Manen, 2017). While these approaches may be of benefit, they are more commonly used in studies at a theoretical level than the empirical studies included in the present review. It is obvious that the data collection method is guided by the research question. However, our review also reveals that the clinical field influences how the data are collected and analysed and that the method may lead to new methods for evaluating clinics.

In the qualitative evaluation checklist guidelines, Patton (2015) emphasizes the importance of the evaluator's knowledge of methodological issues and preparedness to argue for the credibility of the findings. Qualitative evaluations are most often performed in accordance with established methodological guidelines. According to Patton (2015), the quality of qualitative data and analysis depends on skilful interviews, systematic and rigorous observations as well as the sensitivity and integrity of the evaluator (Patton, 2015).

Our review detected that content analysis is common. According to Graneheim, Lindgren, and Lundman (2017), qualitative content analysis typically focuses on subject and context. It emphasizes variation and offers opportunities to perform a manifest descriptive and latent interpretative content analysis (Graneheim & Lundman, 2004). Research using qualitative content analysis is grounded in ontological assumptions, epistemology and methodology. It is important to be aware that the ontological assumptions are open and may vary according to the researchers' standpoint. Another explicit issue is that the epistemological basis of qualitative content analysis should guide the way that data are interpreted: as cocreations of the interviewee and the interviewer. Furthermore, the interpretation method is viewed as a cocreation of the researchers and the text. Graneheim et al. (2017) state that one methodological issue is the difficulties involved in keeping the levels of abstraction and degree of interpretation logical and congruent throughout the analysis and presentation (Graneheim et al., 2017).

In the 1990s, Sandelowski (1996) viewed qualitative methods as the antithesis of clinical research and “far removed from the immediate practical aims of intervention studies and nursing practice” (Sandelowski, 1996, p. 359). However, today we see that such methods not only benefit clinical studies, but are needed to explore, illuminate and describe the variation in the phenomenon to evaluate nursing interventions in their real‐life contexts. Therefore, we believe that the vast number of different methods in the selected articles needs to be outlined and further developed so that such methods will become more common when evaluating in different clinical contexts.

From the *intervention perspective*, the included articles are based on complex interventions (Mohler et al., 2012). Qualitative evaluations seem appropriate when knowledge about the process of testing tools or information about established programmes is needed. It appears to be correct to evaluate any type of intervention qualitatively if the aim is the above‐mentioned knowledge. This supports arguments that the type of evaluative approach is decided by the research question, not the type of intervention.

Another important aspect is whether the intervention is designed ahead of the actual project or whether existing methods or models are to be evaluated. The former adheres to an inductive approach—when the evaluation looks for knowledge derived from the actual practice. According to Graneheim et al. (2017), such an approach is data‐ or text‐driven and characterized by a search for patterns through similarities and differences. This type of analysis is described in categories and/or themes, and the levels of abstraction and interpretation vary. Using the inductive approach, the researcher moves “from the data to a theoretical understanding—from the concrete and specific to the abstract and general” (Graneheim et al., 2017). One important issue that must be addressed when employing an inductive approach is the researchers' pre‐understanding. The question that arises is whether the inductive approach is merely a result of the researchers' pre‐understanding of the studied phenomena. The challenge, according to Graneheim et al. (2017), is to avoid surface descriptions and general summaries when using an inductive approach. A deductive model is employed when data are interpreted through concepts, a model or a theory, and implications about the studied phenomenon are tested against the collected data. In these designs, the researchers move explicitly from theory to data. The challenge, according to Graneheim et al. (2017), is to avoid formulating categories that are exclusively based on established theory or models and the handling of left‐over data. The latter occurs when data are found that do not fit the explanatory model (Graneheim et al., 2017).

The articles included in the present review provide a detailed description of the intervention they evaluated. According to Michie et al. (2009), formal documentation describing the content and delivery of an intervention will help to inform about what to teach new practitioners, how to transform or reorganize healthcare processes and what to include in the assessment of practitioner performance. These are all key features of successful implementation (Michie et al., 2009).

Characteristics of those who deliver the intervention and characteristics that make interventions complex are the different professional categories or varying organizational levels targeted by the intervention (context of the intervention) and/or a need to tailor the intervention to specific settings (flexibility of the intervention) (Mohler et al., 2012). Despite that one narrow inclusion criterion focuses on nursing interventions in a clinical context, we typically find a combination of multiple professional categories delivering nursing interventions in the included articles. Michie et al. (2009) state that description of the characteristics of the setting and of those who deliver an intervention is essential for replicating an implementation strategy.

Intervention level activity is presented as high‐level activity with multiple phases and settings. The need to tailor the intervention to specific settings seems to be the most complex component in the included articles as the evaluations were performed in a natural setting and developed by an actual need in the clinics.

Central questions in the field of evaluating complex interventions are how these interventions work in clinical practice? What are their active components? And are they effective? The answers to such questions will enable new and more effective interventions across multidisciplinary teams in live practice (Michie & Abraham, 2004). The Criteria for Reporting the Development and Evaluation of Complex Interventions in healthcare (CReDECI) may be of use for addressing evaluation (Craig et al., 2008). In contrast to most reporting guidelines, the CReDECI does not offer criteria for a specific study design, but on the process of developing, piloting and evaluating complex interventions (Craig et al., 2008).

Planning is crucial for the implementation of an intervention. According to Morse (Morse et al., 2000), by examining current practice by means of QOA, researchers can contribute to generating increased clinical knowledge. This kind of evaluation can provide a detailed description of local processes in an intervention programme. Morse et al. (2000) claims that QOA may bridge the gap between research and practice. The same could probably be said about the qualitative evaluation method, as it may bring nursing research and practice closer together, and qualitative research methods more accurately describe complex nursing practice. Furthermore, Morse et al. (2000) emphasizes that as nursing is a practice‐based discipline, the development of QOA methodology is critical. We genuinely believe that the same applies to the qualitative evaluation method, which often highlights experiences of a process. As nursing practice is comprehensive and individual, these important characteristics should be emphasized when evaluating it.

The implementation method requires thorough planning, and we assume that such planning is common in clinical nursing. However, the planning of the evaluation seems to be less important compared with the planning of the actual implementation. This may be a result of a dynamic, real‐life situation, which is very much dependent on resources. However, if a new intervention is not evaluated, how will we know what effect it has? We assume that qualitative evaluation is performed at a clinical level—those who receive the intervention are observed and asked at an open level: what was your experience of this intervention? We suggest that these evaluations should be systemized; the responses to open‐ended questions can be collected and analysed with the aim of improving practice. Continuous evaluation during the implementation process is crucial for success.

### 4.1 Strengths and limitations

The strengths and limitations were assessed by the Confidence in the Evidence from Reviews of Qualitative Research Approach (CERQual) (Lewin et al., 2015), which helps assess the confidence in qualitative reviews. CERQual comprises four components, which contribute to assessment of confidence: methodological considerations, relevance, coherence and adequacy of data. We believe that we have thoroughly described the relevance, coherence and adequacy of the data by documenting the review process, the body of evidence and outlining the primary studies. The methodological considerations are the extent to which potential problems in the design are reflected on. The five nurse researchers who conducted the/present review worked in different areas at a University hospital on the West coast of Norway and represent different clinical nursing contexts. We consider this a strength, as we based the analysis and discussion section on rich and deep reflection resulting in the understanding of the review question.

Despite that mixed method evaluations are available, the present review only included qualitative studies. Such a design would illuminate other aspects of evaluation than/that were not a part of the present study.

## 5 CONCLUSION

This review presents a summary of different ways to perform qualitative evaluation in a range of clinical nursing areas and illuminates the complexity involved in evaluation of interventions in naturalistic settings. To the best of our knowledge, no previous review has focused on qualitative evaluation of the implementation of nursing interventions.

The review highlights the fact that to be able to say anything about the needs of nursing in the health field, we must evaluate how nursing functions and nurses act. When caring for the individual patient, qualitative methods are a natural choice for revealing the unique and specific qualities of the experiences of the individual nursing context.

## CONFLICT OF INTEREST

All authors declare that there are no conflicts of interest with regard to this study.

## AUTHOR CONTRIBUTIONS

KR was responsible for writing the manuscript. All authors contributed to the critical revision of the intellectual content, provided feedback on the draft manuscript and approved the final version. They all adhered to the criteria pertaining to roles and responsibilities in the research process recommended by the International Committee of Medical Journal Editors (ICMJE) (http://www.icmje.org/recommendations).

6

**Table 2:** Overview of identified factors

Main theme: Challenging complexity by evaluating qualitatively						
Themes	Evaluating the implementation process			Evaluating improvements brought about by the programme		
Categories	Different types of designs	Data characteristics and context	Types and models of analysis	Clinical benefits	Types of intervention	Characteristics of those who deliver the intervention

## Supporting information

Appendix I: Flow diagram

Appendix II: CASP checklist
