# Supplementary material for: Qualitative evaluation in nursing interventions—A review of the literature
Source: Nurs Open. 2020 Jun 2;7(5):1285–98. doi: 10.1002/nop2.519 (PMC7424442; doi:10.1002/nop2.519)
Supplement: Supplementary file 1 — Appendix I: Flow diagram [file NOP2-7-1285-s001.docx]

APPENDIX II

The Critical Appraisal Skills Programme ([CASP, 2013](#_ENREF_5)) quality assessment of the studies; a methodological checklist of key criteria relevant to qualitative studies. Overview of the results from the included papers based on questions 1-10 from the CASP checklist. Example of assessment: Y: yes, N: no, U: unclear, H: high L: Low

| Author/title: Luker et al**.,** 2016 | Y | U | N | H | M | L |
| --- | --- | --- | --- | --- | --- | --- |
| 1. Was there a clear statement of the aims of the research? | * |  |  |  |  |  |
| 2. Is a qualitative methodology appropriate? | * |  |  |  |  |  |
| 3. Was the research design appropriate to address the aims of the research? | * |  |  |  |  |  |
| 4. Was the recruitment strategy appropriate to the aims of the research? | * |  |  |  |  |  |
| 5. Was the data collected in a way that addressed the research issue? | * |  |  |  |  |  |
| 6. Has the relationship between researcher and participants been adequately considered? | * |  |  |  |  |  |
| 7. Have ethical issues been taken into consideration? | * |  |  |  |  |  |
| 8. Was the data analysis sufficiently rigorous? | * |  |  |  |  |  |
| 9. Is there a clear statement of findings? | * |  |  |  |  |  |
| 10. How valuable is the research? |  |  |  | * |  |  |

|  | **Study** | **1** | **2** | **3** | **4** | **5** | **6** | **7** | **8** | **9** | **10** |
| --- | --- | --- | --- | --- | --- | --- | --- | --- | --- | --- | --- |
| 1 | Baron et al., 2018 | N | Y | Y | Y | Y | Y | N | Y | Y | H |
| 2 | Bolmsjö et al., 2014 | Y | Y | Y | Y | Y | N | N | Y | Y | H |
| 3 | Clignet et al., 2016 | Y | Y | Y | Y | Y | U | U | Y | Y | H |
| 4 | Davidsson & Swanson, 2018 | Y | Y | Y | Y | Y | N | Y | Y | Y | H |
| 5 | Furler et al., 2014 | Y | Y | Y | Y | Y | N | Y | Y | Y | H |
| 6 | Graves et al., 2016 | Y | Y | Y | Y | Y | N | Y | Y | Y | H |
| 7 | Hahne et al., 2017 | Y | Y | Y | Y | Y | Y | U | Y | Y | M |
| 8 | Halcomb et al., 2015 | Y | Y | Y | Y | Y | Y | Y | Y | Y | H |
| 9 | Hanifa et al., 2018 | Y | Y | Y | Y | Y | Y | Y | Y | Y | M |
| 10 | Helmle et al., 2018 | Y | Y | Y | Y | Y | Y | Y | Y | Y | H |
| 11 | Hill et al., 2016 | Y | U | Y | Y | Y | U | Y | Y | Y | H |
| 12 | Iyer et al., 2015 | Y | Y | Y | Y | Y | N | Y | Y | Y | H |
| 13 | Kang et al., 2017 | Y | Y | Y | Y | Y | N | Y | Y | Y | M |
| 14 | Luker et al., 2016 | Y | Y | Y | Y | Y | Y | Y | Y | Y | H |
| 15 | Søderlund et al., 2016 | Y | Y | Y | Y | Y | Y | Y | Y | Y | H |
