# Supplementary material for: Qualitative evaluation in nursing interventions—A review of the literature
Source: Nurs Open. 2020 Jun 2;7(5):1285–98. doi: 10.1002/nop2.519 (PMC7424442; doi:10.1002/nop2.519)
Supplement: Supplementary file 2 — Appendix II: CASP checklist [file NOP2-7-1285-s002.doc]

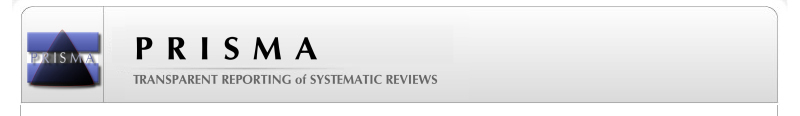
**Appendix I: PRISMA 2009 Flow Diagram**

**Screening**

**Included**

**Eligibility**

**Identification**

PsychINFO (20), MEDLINE (27) and Embase (25)

(n = 72)

Additional records identified through other sources, CINAHL (n=29)

(n = 0)

Records after duplicates removed
(n = 40)

Records screened
(n = 45)

Records excluded due to non-qualitative method

(n = 13)

Full-text articles assessed for eligibility
(n = 34)

Full-text articles excluded, with reasons
(n = 6)

Studies included in qualitative synthesis
(n = 15)

Records after duplicates removed
(n = 15)

Full-text articles excluded, published 2013 and earlier
(n = 13)
